# *Oligoflexus tunisiensis* gen. nov., sp. nov., a Gram-negative, aerobic, filamentous bacterium of a novel proteobacterial lineage, and description of *Oligoflexaceae* fam. nov., *Oligoflexales* ord. nov. and *Oligoflexia* classis nov.

**DOI:** 10.1099/ijs.0.060798-0

**Published:** 2014-10

**Authors:** Ryosuke Nakai, Miyuki Nishijima, Nozomi Tazato, Yutaka Handa, Fatma Karray, Sami Sayadi, Hiroko Isoda, Takeshi Naganuma

**Affiliations:** 1 Graduate School of Biosphere Science, Hiroshima University, 1-4-4 Kagamiyama, Higashi-hiroshima, Hiroshima 739-8528, Japan; 2 Research Fellow of the Japan Society for the Promotion of Science, Chiyoda-ku, Tokyo 102-8471, Japan; 3 Technical Department, TechnoSuruga Laboratory Co., Ltd., 330 Nagasaki, Shimizu-ku, Shizuoka 424-0065, Japan; 4 Centre of Biotechnology at Sfax, P.O. Box 1177, 3018 Sfax, Tunisia; 5 Alliance for Research on North Africa (ARENA), University of Tsukuba, 1-1-1 Tennoudai, Tsukuba, Ibaraki 305-8572, Japan

## Abstract

A phylogenetically novel proteobacterium, strain Shr3^T^, was isolated from sand gravels collected from the eastern margin of the Sahara Desert. The isolation strategy targeted bacteria filterable through 0.2-µm-pore-size filters. Strain Shr3^T^ was determined to be a Gram-negative, aerobic, non-motile, filamentous bacterium. Oxidase and catalase reactions were positive. Strain Shr3^T^ showed growth on R2A medium, but poor or no growth on nutrient agar, trypticase soy agar and standard method agar. The major isoprenoid quinone was menaquinone-7. The dominant cellular fatty acids detected were C_16 : 1_ω5*c* and C_16 : 0_, and the primary hydroxy acid present was C_12 : 0_ 3-OH. The DNA G+C content was 54.0 mol%. Phylogenetic analysis based on 16S rRNA gene sequences revealed that strain Shr3^T^ was affiliated with an uncultivated lineage of the phylum *Proteobacteria*; the nearest known type strain, with 83 % sequence similarity, was *Desulfomicrobium orale* DSM 12838^T^ in the class *Deltaproteobacteria*. The isolate and closely related environmental clones formed a novel class-level clade in the phylum *Proteobacteria* with high bootstrap support (96–99 %). Based on these results, the novel class *Oligoflexia* classis nov. in the phylum *Proteobacteria* and the novel genus and species *Oligoflexus tunisiensis* gen. nov., sp. nov. are proposed for strain Shr3^T^, the first cultivated representative of the *Oligoflexia*. The type strain of *Oligoflexus tunisiensis* is Shr3^T^ ( = JCM 16864^T^ = NCIMB 14846^T^). We also propose the subordinate taxa *Oligoflexales* ord. nov. and *Oligoflexaceae* fam. nov. in the class *Oligoflexia*.

Culture-independent molecular approaches have revealed that microbial life is astoundingly diverse, and that most prokaryotic microbes have not yet been cultivated (Amann *et al.*, 1995; Rappé & Giovannoni, 2003). While high-throughput sequencing may reveal a comprehensive picture of microbial community structure, directed cultivation still has an important role in describing microbial phenotype, metabolism and physiology. To obtain yet-to-be cultured microorganisms, we have focused on the fraction filterable by 0.2-µm-pore-size filters (Elsaied *et al.*, 2001; Nakai *et al.*, 2013).

In scientific research and many medical and industrial applications, sterile filtration with 0.2-µm filters is a commonly used method of removing microorganisms. Several studies have reported, however, that previously uncultured bacteria can be isolated from 0.2-µm-filtered freshwater or seawater samples (e.g. Elsaied *et al.*, 2001; Hahn, 2004; Hahn *et al.*, 2004). These results are attributed to the effective removal by filtration of readily cultivable, fast-growing bacteria that are able to overgrow slow-growing bacteria (Hahn *et al.*, 2004). Community structure and potential function of 0.2-µm-filterable populations in various environments have recently been investigated using culture-independent methods (Miyoshi *et al.*, 2005; Naganuma *et al.*, 2007; Nakai *et al.*, 2011). For example, 0.2-µm-filterable (0.1-µm-captured) phylotypes obtained from deep terrestrial aquifers included members of the candidate divisions OD1 and OP11, representing novel phylum-level lineages (Miyoshi *et al.*, 2005). In addition, filterable populations in deep-sea hydrothermal fluid contained novel phylotypes (Naganuma *et al.*, 2007) and gene sequences pertinent to membrane functions (Nakai *et al.*, 2011). It therefore appears that microbiologists may have largely underestimated the diversity of the 0.2-µm-filterable fraction.

While surveying 0.2-µm-filterable bacteria, we isolated a bacterium, designated strain Shr3^T^, from sand gravels collected from the eastern margin of the Sahara Desert (Nakai *et al.*, 2013). Phylogenetic analyses based on 16S rRNA gene sequences indicated that strain Shr3^T^ belongs to a novel lineage of phylum *Proteobacteria*. In this study, we characterized strain Shr3^T^ by using a polyphasic approach. Based on our results, we propose the name *Oligoflexus tunisiensis* gen. nov., sp. nov. to accommodate the isolate, and define a novel class, *Oligoflexia* classis nov., within the phylum *Proteobacteria*.

Strain Shr3^T^ was originally isolated from sand gravels collected in December 2008 in Matmata (33° 31′ N 9° 57′ E) on the eastern margin of the Sahara Desert in the Republic of Tunisia. Isolation of filterable bacteria was conducted as described previously (Elsaied *et al.*, 2001), with some modifications. The sample was suspended in PBS containing 8 g NaCl, 1.1 g Na_2_HPO_4_, 0.2 g KCl and 0.2 g KH_2_PO_4_ in 1 l distilled water. The resulting suspension was filtered threefold through sterilized 0.2-µm filter units (Advantec). In our study, three filters were connected serially for secured filtration. The filtrate was then added aseptically to 5× R2A liquid medium (1× concentration: 0.5 g yeast extract, 0.5 g peptone, 0.5 g casamino acids, 0.5 g glucose, 0.5 g soluble starch, 0.3 g sodium pyruvate, 0.3 g K_2_HPO_4_, 0.05 g MgSO_4_ · 7H_2_O in 1 l distilled water; pH 7.2) to obtain a final concentration of 1×. R2A medium is a relatively low-nutrient medium used for cultivation of environmental microorganisms (Reasoner & Geldreich, 1985). This culture liquid was incubated in the dark at room temperature. The turbid culture was subsequently spread onto 1.5 % agar containing identical ingredients of R2A liquid medium and incubated for colony formation at room temperature. A single colony was selected and inoculated onto fresh R2A agar medium to purify a colony-forming strain. This procedure was repeated at least three times to ensure reliable colony purification.

Colonies of strain Shr3^T^ were circular to irregular, faintly pale yellow and 1.5–2.0 mm in diameter on plates of R2A agar DAIGO (Nihon Pharmaceutical) (unless otherwise described, this agar medium was used for subsequent experiments and denoted as R2A) after 5 days at 25 °C. Cell morphology was examined by light microscopy (BX50; Olympus) and transmission electron microscopy (TEM). Cells for light microscopic observation were grown on R2A for 5 days at 25 °C. Cells were filamentous (width 0.4–0.8 µm) or elongated spindle-shaped cells, some of which exhibited a thin and flexible spiral form similar to a spirillum (Fig. 1).

**Fig. 1.**
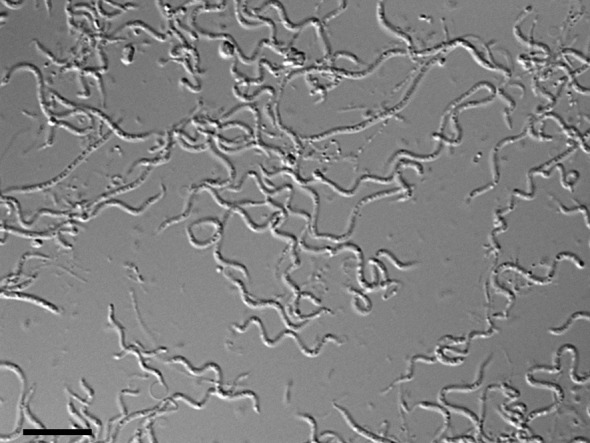
Light micrograph of cells of strain Shr3^T^. Filamentous, elongated fusiform and spiral cells were observed. Cells were grown on R2A for 5 days at 25 °C. Observations were made using differential interference contrast microscopy. Bar, 10 µm.

Ultrathin sectioning and TEM were carried out for observation of intracellular construction. Cells grown on R2A for 7 days at 25 °C were fixed with 2 % glutaraldehyde in 0.1 M phosphate buffer (pre-fixation), followed by post-fixation using osmium tetroxide. After dehydration in an ethanol series, the sample was embedded with resin and sectioned. Following staining with uranyl acetate and modified Sato’s lead staining solution (Hanaichi *et al.*, 1986), the specimen was examined using a JEM-1200EX transmission electron microscope (JEOL). Cells of strain Shr3^T^ were found to possess a Gram-negative cell envelope, with cytoplasmic and outer membranes readily visible (Fig. 2). Elongated cells or long rod-shaped cells (more than 10 µm long) were observed in the sectioned samples. The cells appeared to be very weak or to have a weak cell wall with little resistance to osmotic pressure; although the cells were similar in this regard to spirilla, no flagella-like structures were observed. Cells grown on R2A were wider than the 0.2-µm pores of the filter. The cells appeared to be flexible and slender under microscopic observation, and this may lead to their filterability through a 0.2-µm-pore-size filter. Alternatively, it may be that, under native growth conditions, cells of this bacterium are thinner than under cultivation on R2A. Cells reproduced by binary fission, although we did not observe budding morphology. Spore formation was not observed. Cells contained many low electron-dense particles (granules) (Fig. 2), with many nucleoid and ribosome particles present. Neisser and DAPI staining were performed to test for the presence of polyphosphate inclusions. Neisser-stained cells and DAPI-stained cells were observed under a light microscope and a fluorescence microscope (AX80; Olympus), respectively. Polyphosphate inclusions were not observed using either staining method.

**Fig. 2.**
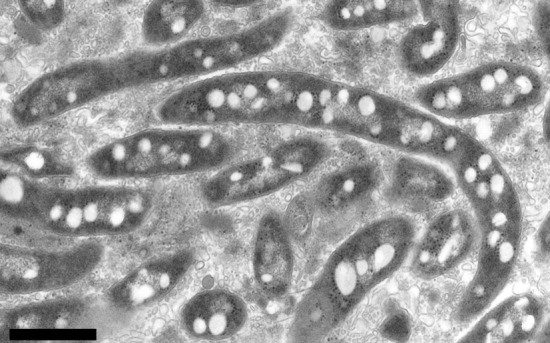
Transmission electron micrograph of cells of strain Shr3^T^. Many low electron-dense particles were observed. Cells were grown on R2A for 7 days at 25 °C. Bar, 1 µm.

Conventional taxonomic features were determined according to procedures described by Cowan and Steel as revised by Barrow & Feltham (1993). Strain Shr3^T^ was cultivated on R2A at 25 °C, unless otherwise stated. Most characteristics of strain Shr3^T^ could not be measured because of the low nutrient conditions required by the isolate. To assess carbohydrate utilization, we tried two different basal media: M70 medium (Veron, 1975) and one described by Palleroni & Doudoroff (1972). Carbohydrates were added to each of these basal media to give a final concentration of 0.1 % (w/v). Anaerobic cultivation was performed using an Anaero Pack system (Mitsubishi Gas Chemical). Sulfate reduction ability of strain Shr3^T^ was examined using modified ISA medium (Mara & Williams, 1970). We also used an API 20NE kit (bioMérieux) to assess biochemical characteristics and carbohydrate utilization, and conducted an API ZYM test (bioMérieux) to evaluate enzyme activities. In addition to Neisser and DAPI staining as described above, polyhydroxybutyrate (PHB) accumulation was tested.

The isolate was Gram-negative, oxidase- and catalase-positive and non-spore-forming. Motility and flagellation of cells were not observed. Strain Shr3^T^ did not grow under anaerobic conditions. It grew in low-nutrient media such as R2A, but poorly or not at all on nutrient agar (Oxoid), standard method agar (Nissui) and trypticase soy agar (Becton Dickinson). Even on R2A, the cells exhibited comparatively slow growth, with 3–5 days required before colonies were observable with the naked eye. Strain Shr3^T^ did not require vitamins for growth. The isolate was able to grow in the presence of 0.5 % (w/v) NaCl, but not at 1 % or higher concentrations. The pH range for growth was 7.0–9.5, with an optimum range of pH 7.0–8.0. Strain Shr3^T^ grew at 20–37 °C, with an optimum temperature of 25–30 °C; no growth was observed below 15 °C or above 40 °C. Tested carbohydrates were not utilized in either basal medium. Based on API 20NE results, strain Shr3^T^ displayed a positive reaction for gelatin liquefaction, but was negative for the other tests. Sulfate reduction was not observed. Cells swollen by accumulated PHB were not observed when grown on PHB-containing medium. Particles stained by Sudan black B were observed inside the cells, however, when cells were grown on R2A or PHB-containing medium. Other detailed phenotypic features of the isolate are given in the species description.

To analyse the isoprenoid quinone system of the isolate, cells grown on R2A for 4 days at 30 °C were used. Quinone extraction and measurement was performed by HPLC (Waters) according to Nishijima *et al.* (1997). Menaquinone-7 (MK-7) was the only quinone detected. The cellular fatty acid composition was analysed using cells grown on R2A for 8 days at 25 °C. Cellular fatty acid methyl esters were prepared and analysed by gas chromatography (HP6890 series GC system; Hewlett Packard) using the MIDI Microbial Identification system version 4.5, with profiles compared against the Sherlock System 4.5 database (Microbial ID; MIDI Inc.). The dominant fatty acids of strain Shr3^T^ were C_16 : 1_ω5*c* (65.7 %) and C_16 : 0_ (27.5 %); the major hydroxy acid was C_12 : 0_ 3-OH (1.3 %). Other minor components are shown in Table S1 (available in the online Supplementary Material).

Genomic DNA was extracted and purified as described by Nishijima *et al.* (2009). The DNA G+C content of the isolate was determined as described by Nishijima *et al.* (2009). Briefly, extracted genomic DNA was digested to nucleotides with nuclease P1 using a DNA-GC kit (Seikagaku Kogyo) according to the procedure described by Katayama-Fujimura *et al.* (1984). The G+C content of the digested DNA was then determined by HPLC (LC-10; Shimadzu) with an RP Aqueous column (4.6×250 mm; Nomura Chemical) and a UV-Vis spectrophotometric detector (SPD-10AV; Shimadzu) at 270 nm. The DNA G+C content of strain Shr3^T^ was 54.0 mol%.

Sequencing and phylogenetic analysis of the 16S rRNA gene of the isolate were performed as described previously (Tazato *et al.*, 2012). The 16S rRNA gene sequence of strain Shr3^T^ (1458 bp) was compared with known sequences in the DDBJ nucleotide database (http://www.ddbj.nig.ac.jp/) using a blast search (Altschul *et al.*, 1997). The nearest known type strain, with 83 % sequence similarity, was *Desulfomicrobium orale* DSM 12838^T^ in the class *Deltaproteobacteria*. The closest environmental sequence, with 98.3 % similarity, came from rice paddy soil (DDBJ accession no. AB486128; Ishii *et al.*, 2009). In the phylogenetic tree constructed using the neighbour-joining method, strain Shr3^T^ did not cluster with any of the known classes of phylum *Proteobacteria*. The isolate and closely related environmental clones formed a well-supported (99 % bootstrap support) clade nested deeply within the *Proteobacteria* (Fig. 3). In trees reconstructed by the maximum-likelihood, minimum evolution and maximum-parsimony methods (Figs. S1–S3) with the same sequence dataset as the neighbour-joining tree, the isolate was also grouped in a similar clade. This clade, having 96–99 % bootstrap support, included environmental clone sequences recovered from various samples, including soil, desert sands, plant rhizospheres, earthworm intestines, wastewater treatment biofilms, glacier ice, seawater and green algal surfaces, all sharing 95.9–98.3 % 16S rRNA gene sequence similarity. These results suggest that the novel bacterial lineage that includes strain Shr3^T^ is ubiquitous in the environment.

**Fig. 3.**
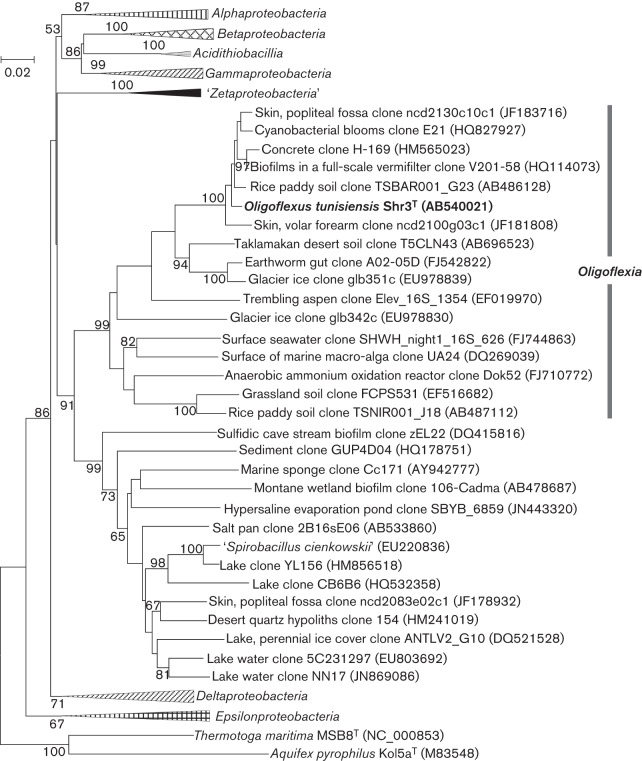
Phylogenetic relationships of strain Shr3^T^, related environmental clones and known groups in the phylum *Proteobacteria* based on 16S rRNA gene sequences. The tree, constructed using the neighbour-joining method, is based on a comparison of 1124 nucleotide positions. Bootstrap values >50 %, expressed as percentages of 1000 replicates, are shown above and below branches. Bar, 0.02 substitutions per nucleotide position. Trees reconstructed by the maximum-likelihood, minimum evolution and maximum-parsimony methods are available as Figs S1–S3.

Although strain Shr3^T^ could not be characterized fully because of its restrictive culture conditions, measured physiological and biochemical features were typical of the phylum *Proteobacteria*. Its cell shape was somewhat unusual, however, as it exhibited filamentous, curved rod and spirillum-like spiral forms.

The major isoprenoid quinone found in the isolate was MK-7; ubiquinones and other quinones were not detected. Although ubiquinones generally predominate in aerobic bacteria belonging to the classes *Alphaproteobacteria*, *Betaproteobacteria* and *Gammaproteobacteria* (Collins & Jones, 1981; Brenner *et al.*, 2005a, b), some anaerobic bacteria, including facultatively anaerobic bacteria, in those classes and in the *Deltaproteobacteria* and *Epsilonproteobacteria* produce menaquinones as their dominant quinone (Collins & Jones, 1981; Brenner *et al.*, 2005a, b). In the *Deltaproteobacteria*, two groups of bacteria produce menaquinones: obligately anaerobic bacteria, which are able to reduce sulfate, and aerobic myxobacteria. With respect to the quinone system, our isolate was thus similar to myxobacteria in the *Deltaproteobacteria*. The major fatty acids in our isolate differed from those of other known proteobacteria in regard to the proportions of C_16 : 1_ω5*c* and C_16 : 0_. These two fatty acids constituted >93 % of the detected cellular fatty acids. The fatty acid composition of strain Shr3^T^ was most similar to that of myxobacteria, of which C_16 : 1_ω5*c* is a major component, corresponding to >40 % of detected fatty acids (Kearns *et al.*, 2001; Shimkets *et al.*, 2006).

Although chemotaxonomic examination suggested that strain Shr3^T^ was comparatively similar to members of the class *Deltaproteobacteria*, the isolate was unable to reduce sulfate or to carry out anaerobic growth under our experimental conditions, in contrast to results observed for sulfate-reducing deltaproteobacteria. Furthermore, no myxobacteria-like cell cycle was observed in Shr3^T^.

The phylum *Proteobacteria* currently comprises five classes, with two additional classes, ‘*Zetaproteobacteria*’ and *Acidithiobacillia*, proposed recently by Emerson *et al.* (2007) and Williams & Kelly (2013), respectively. Based on our phylogenetic analysis, strain Shr3^T^ is distantly related to other known classes in the *Proteobacteria*, forming instead a novel, well-supported clade along with closely related clones. Although the phylogenetic analyses also suggested that the deep-branching but not yet cultivated deltaproteobacterium ‘*Spirobacillus cienkowskii*’ (Rodrigues *et al.*, 2008) and related environmental sequences form a new class-level clade (Fig. 3), further evaluation requires the establishment of a cultured strain and an analysis of its physiological and chemotaxonomic characteristics.

Based on the results of the polyphasic examination described above, strain Shr3^T^ is a novel and distinctive bacterium in the phylum *Proteobacteria*. Consequently, we propose a novel genus and species, *Oligoflexus tunisiensis* gen. nov., sp. nov., to accommodate the isolate, and create a novel class, *Oligoflexia* classis nov., with strain Shr3^T^ as its sole cultured representative. Taxonomic ranks between class and genus are also proposed.

## Description of *Oligoflexus* gen. nov.

*Oligoflexus* (O.li.go.fle′xus. Gr. adj. *oligos* little, few; L. part. adj. *flexus* bent, curved; N.L. masc. n. *Oligoflexus* flexible utilizer of few substrates).

Gram-negative. Cells are non-motile and non-spore-forming. Aerobic. Cell shape is pleiomorphic, such as a filamentous, flexible, long fusiform shape or a helical shape similar to spirilla. Cells grow under low nutrient conditions. MK-7 is produced as a major quinone under aerobic cultivation conditions. The major cellular fatty acids are C_16 : 1_ω5*c* and C_16 : 0_. The DNA G+C content of the type strain of the type species is 54.0 mol%. The type species is *Oligoflexus tunisiensis*.

## Description of *Oligoflexus tunisiensis* sp. nov.

*Oligoflexus tunisiensis* (tu.ni.si.en′sis. N.L. masc. n. *tunisiensis* pertaining to Tunisia, where the type strain was isolated).

This species has all the characteristics that define the genus, in addition to those described below. Catalase- and oxidase-positive. Non-pigmented. Does not require growth factors. Grows under low nutrient conditions, such as on R2A medium, but exhibits poor or no growth on nutrient agar, trypticase soy agar and standard method agar. Growth rate is slow. Colonies are circular to irregular, low-convex, undulate, pale creamy white and opaque, 1.5–2.0 mm in diameter after 3–5 days of incubation on R2A at 25 °C. Cells are 0.4–0.8 µm wide on R2A. Neisser staining is negative. Sudan black B-stained particles are observed. Non-halophilic and mesophilic. Grows at NaCl concentrations of <1.0 % (w/v). Growth occurs at 20–37 °C, with an optimum temperature of 25–30 °C, and at pH 7.0–9.5, with an optimum at pH 7.0–8.0. Sulfate reduction is not observed. Tested carbohydrates (D-glucose, l-arabinose, d-mannose, *N*-acetyl-d-glucosamine, d-maltose, lactose, *myo*-inositol, adonitol, d-sorbitol, d-mannitol, d-trehalose, glycerol, salicin, l-rhamnose, sucrose, d-cellobiose, citrate, fumarate and malate) are not utilized in two tested basal media. Based on the API 20NE test, positive for gelatin liquefaction, and negative for nitrate reduction, indole production, arginine dihydrolase, urease, aesculin hydrolysis, *p*-nitrophenyl β-d-galactopyranoside hydrolysis and utilization of d-glucose, l-arabinose, d-mannose, d-mannitol, *N*-acetyl-d-glucosamine, d-maltose, potassium gluconate, capric acid, adipic acid, malic acid, trisodium citrate and phenylacetic acid. Enzyme activities of esterase lipase (C8), leucine arylamidase, trypsin, naphthol-AS–BI-phosphohydrolase and α-mannosidase are positive. Alkaline phosphatase, esterase (C4), lipase (C14), valine arylamidase, cystine arylamidase, chymotrypsin, acid phosphatase, α-galactosidase, β-galactosidase, β-glucuronidase, α-glucosidase, β-glucosidase, *N*-acetyl-β-glucosaminidase and α-fucosidase tests are negative (API ZYM). The major hydroxy fatty acid is C_12 : 0_ 3-OH.

The type strain, Shr3^T^ ( = JCM 16864^T^ = NCIMB 14846^T^), was isolated from sand gravels collected from the Sahara Desert in the Republic of Tunisia. The DNA G+C content of the type strain is 54.0 mol%.

## Description of *Oligoflexaceae* fam. nov.

*Oligoflexaceae* (O.li.go.flex.a′ce.ae. N.L. masc. n. *Oligoflexus* type genus of the family; suff. -*aceae* ending to denote a family; N.L. fem. pl. n. *Oligoflexaceae* the family of the genus *Oligoflexus*).

The description is the same as for the genus *Oligoflexus*. The type genus is *Oligoflexus*.

## Description of *Oligoflexales* ord. nov.

*Oligoflexales* (O.li.go.flex.a′les. N.L. masc. n. *Oligoflexus* type genus of the order; suff. -*ales* ending to denote an order; N.L. fem. pl. n. *Oligoflexales* the order of the genus *Oligoflexus*).

The description is the same as for the genus *Oligoflexus*. The type genus is *Oligoflexus*.

## Description of *Oligoflexia* classis nov.

*Oligoflexia* (O.li.go.flex.i.a. N.L. masc. n. *Oligoflexus* type genus of the type order of the class; suff. -*ia* ending to denote a class; N.L. fem. pl. n. *Oligoflexia* the class of the order *Oligoflexales*).

The class is defined on the basis of a phylogenetic analysis of 16S rRNA gene sequences of a single isolated strain and uncultured representatives from various environments. The type order is *Oligoflexales*.
